# Aortopulmonary window due to transcatheter pulmonary valve implantation after arterial switch operation

**DOI:** 10.1186/1749-8090-10-S1-A251

**Published:** 2015-12-16

**Authors:** María-Teresa González-López, Juan-Miguel GilJaurena, José-Luis Zunzunegui-Martínez, Reyes álvarez-García-Rovés, Constancio Medrano-López, Ramón Pérez-Caballero-Martínez, Ana-María Pita-Fernández

**Affiliations:** 1 Paediatric Cardiac Surgery Department, Gregorio Marañón Hospital, Madrid, Spain; 2 Paediatric Cardiology Department, Gregorio Marañón Hospital, Madrid, Spain

## Background/Introduction

Right-sided lesions occur in 5-25% of patients after arterial switch operation (ASO) and percutaneous or surgical treatments are available (although they are not complication-free).

## Aims/Objectives

We present the first reported case of iatrogenic aorto-pulmonary window (APW) due to transcatheter pulmonary valve implantation after ASO and the surgical strategy for repair.

## Method

A 12-year old boy (d-transposition of the great arteries and ASO (LeCompte maneuver) in the neonatal period) was referred for transcatheter pulmonary valve implantation due to pulmonary regurgitation. A 22-mm stent-mounted valved bovine jugular vein graft (Melody valve, Medtronic, Minneapolis) was implanted, but the patient became hemodynamically unstable (pulmonary-to-systemic ratio 1.96). Transesophageal echocardiography (TEE) showed a traumatic APW in the uppermost portion of the ascending aorta. Haemodynamic stability was achieved following closure using a 10-mm Amplatzer-Muscular-VSD-Occluder but he remained symptomatic in the subsequent weeks. TEE showed residual left-to-right shunt in the proximal margin and a covered stent was implanted on the left pulmonary artery (LPA) to deal with the residual shunt, but the Amplatzer was dislocated. He was referred for emergent surgery. He was cooled to 18°C and ventricular fibrillation was achieved. Carbon dioxide field flooding was employed throughout. The circulation was arrested and the right PA was incised and extended toward the LPA. Both percutaneous devices were removed and the APW was closed (Gore-Tex patch). The PA and branches were reconstructed (bypass time 150 and arrest time 23 minutes). TEE showed no residual shunts.

## Results

He had an uneventful recovery and was discharged on 10th postoperative day.

## Discussion/Conclusion

The adherences between the aorta and PA on performing the LeCompte maneuver and the protrusion of the stent-mounted valve into the PA bifurcation may result in an APW. Care should be taken in avoiding Melody valve in patients with ASO and surgical option such as valved conduit is advised when significant pulmonary regurgitation is developed.

## Consent

Written informed consent was obtained from the patient for publication of this abstract and any accompanying images. A copy of the written consent is available for review by the Editor of this journal.

